# National Italian Delphi panel consensus: which measures are indicated to minimize pegylated-asparaginase associated toxicity during treatment of adult acute lymphoblastic leukemia?

**DOI:** 10.1186/s12885-020-07461-5

**Published:** 2020-10-02

**Authors:** Federico Lussana, Paola Minetto, Felicetto Ferrara, Sabina Chiaretti, Giorgina Specchia, Renato Bassan

**Affiliations:** 1 Hematology and Bone Marrow Transplant Unit, Azienda Socio Sanitaria Territoriale Papa Giovanni XXIII, Piazza OMS, 1, 24127 Bergamo, Italy; 2 Clinic of Hematology, Department of Internal Medicine (DiMI), University of Genoa, Genova, Italy; 3 IRCCS, Ospedale Policlinico San Martino, Genova, Italy; 4 Division of Hematology, Cardarelli Hospital, Naples, Italy; 5 Hematology, Department of Translational and Precision Medicine, Sapienza University, Rome, Italy; 6 Department of Emergency and Organ Transplantation (D.E.T.O.), Hematology Section, University of Bari, Bari, Italy; 7 Complex Operative Unit of Haematology, dell’Angelo Hospital and Santissimi Giovanni and Paolo Hospital, Mestre and Venice, Italy

**Keywords:** Pegylated asparaginase, Acute lymphoblastic leukemia, Toxicity

## Abstract

**Background:**

L-asparaginase (L-ASP) is a key component of acute lymphoblastic leukemia (ALL) treatment, but its use in clinical practice raises challenges to clinicians due to a relatively high incidence of drug-related adverse events, mainly in adult patients. In the past years the use of ASP in adult population has been mainly limited due to a poor knowledge of its safety profile and to an approximate management of ASP-related toxicity. Recently the development of pediatric-inspired treatment protocols for adult ALL has led to a wider use of ASP and since 2010 in Italy three national treatment protocols including Pegylated asparaginase (Peg-ASP) have been sequentially developed for adolescents, young adults and adults with Philadelphia-negative (Ph-) ALL.

**Methods:**

With the aim to better understand the approach adopted in Italian centers for the management and prevention of Peg-ASP toxicity in adult ALL and to provide practical, consensus-based recommendations, a board of 6 Italian clinicians, with known expertise in adult ALL, designed 41 consensus statements on current challenges on the management of Peg-ASP associated toxicity. A group of 19 clinical experts in the field then rated these statements using the 5-point Likert-type scale (1 = strongly disagree; 5 = strongly agree).

**Results:**

The main Peg-ASP related issues identified by the board included: 1) clinician’s attitudes; 2) toxicity profile; 3) hypersensitivity reactions; 4) hepatic toxicity; 5) hepatic and/or metabolic toxicity; 6) hemorrhagic/thrombotic toxicity; 7) pancreatitis; 8) metabolic toxicity management and prevention; 9) activity levels monitoring. Overall, participants agreed on most statements, except those addressing the potential contraindications to the treatment with Peg-ASP, such as patients with a diagnosis of chronic liver disease or the subsequent administrations of the drug in patients who had previously developed chemical pancreatitis or severe metabolic toxicity. Participants agreed that adult patients with ALL should receive Peg-Asp because this drug is essential to improve treatment results.

**Conclusions:**

The panel agreed that a critical evaluation of specific risk factors for each patient is crucial in order to reduce the risk of adverse events and specific advices in the management of Peg-ASP toxicities are reported.

## Background

L-asparaginase (L-ASP) is a key component of acute lymphoblastic leukemia (ALL) treatment both in childhood and adult setting [1–3]. Lymphoblastic leukemic cells do not express the enzyme asparagine synthetase, therefore are unable to produce asparagine de novo and depend on extracellular sources. The clinical effectiveness of L-ASP is based upon the depletion of circulating asparagine, since a sustained depletion of asparagine may lead to leukemic cell death [4]. Most of our knowledge about L-ASP activity and toxicity profile derives from the pediatric experience which has extensively incorporated L-ASP into ALL treatment protocols over the last 50 years [5]. On the contrary, the relatively higher incidence of drug-related adverse events in adult patients has limited its use in adults. Only in the last few decades the development of pediatric-inspired treatment protocols for adult ALL has led to a wider use of L-ASP in these patients, confirming its significant anti-leukemic activity also in the adult setting [6–8].

Pegylated asparaginase (Peg-ASP) gives the advantage of a more favorable pharmacokinetic and immunologic profile, that allows to achieve 2–3 weeks of serum enzymatic activity after a single drug administration and a significant reduction of L-ASP neutralizing antibodies development [9–13]. The toxicity profile of Peg-ASP has been proven to be similar to that of the native *E. coli* formulation and therefore in 2006 Peg-ASP has received FDA approval for the first-line treatment of ALL. However, in contrast to the pediatric setting, the optimal schedule and safety profile of Peg-ASP administration in the adult population have not yet been clearly established. Moreover, the longer half-life conferred by pegylation raises concerns about the management of a worrisome long-lasting toxicity during the back-ground chemotherapy schedule.

It is known that the risk of developing ASP-related toxicities is age-dependent with a proportional increase in incidence and severity. In addition, pharmacokinetic studies suggest that adolescent and adult ALL patients have a lower rate of ASP clearance [14, 15]. However, serum enzymatic activity monitoring has not been routinely performed and reliable pharmacokinetic data on the use of Peg-ASP in the adult population are lacking.

The most serious adverse effects associated with ASP in adult ALL patients are hepatotoxicity, pancreatitis, hyperglycemia and hypertriglyceridemia, thrombosis and alterations of the hemocoagulative parameters and hypersensitivity [16]. In this manuscript we refer to the main ASP-associated toxicities using the definition and grade of severity according to the Common Terminology Criteria for Adverse Events (CTCAE) version5.0 [17].

The development of severe toxicities can lead to significant delays in chemotherapy administration, impairing the efficacy of the whole antileukemic treatment and can be potentially life-threatening especially when associated with severe neutropenia and infectious complications [18]. All in all, it is mandatory to identify patients at high risk of developing high-grade ASP-related toxicities for an effective and homogeneous approach to the prevention and management of these toxicities in order to improve the safety and efficacy of ASP treatment in adult ALL. This is a very relevant clinical issue, because modern pediatric-inspired ASP-containing regimens yielded significantly better survival results than ASP-free regimens in Ph- ALL, at least in adolescent and young adults and up to an age of 55 years [19–21]. The most notable experience with Peg-ASP in adult ALL has been reported by the German study group [7]. In a recent update on 2019 total patients aged 15–55 years and treated in the 07/03 study, the use of Peg-ASP during induction/consolidation (7 total doses) led to progressively better results in the standard risk group, with a 5-year CR rate of 63 and 77% using Peg-ASP at 500–1000 IU/m^2^ (cohort 1, *n* = 437) or at 2000 IU/m^2^ (cohort 2, *n* = 300), respectively (*P* = 0.002) [22].

Based on this background and since three national treatment protocols including Peg-ASP have been sequentially developed from 2010 onwards in Italy for Ph- ALL in adolescents and young adults (AYA, age range 18–35 years: Gruppo Italiano Malattie Ematologiche dell’Adulto (GIMEMA) LAL 1308) [23] and adult ALL (age range 18–65 years; GIMEMA LAL 2317 and LAL1913) [24, 25], the present work aimed to critically examine the approach adopted in Italian centers to manage Peg-ASP toxicity in adult ALL and to provide practical, consensus-based recommendations from a national panel of ALL Experts.

## Methods

### Literature revision and the Delphi method

A group of six Italian clinicians (thereafter referred to as the board), with known interest and high skills in treating adult ALL, aimed at reaching a consensus on current challenges related to the management of Peg-ASP associated toxicity in adult patients with ALL, by adopting the Delphi method, a well-established methodology used in the scientific field [26–28]. After a careful review of the scientific literature, the board identified the following four topics currently lacking a clinical consensus and elaborated a Delphi questionnaire:

- Asparaginase use and formulation type in the different phases of ALL therapy in adults;
- Pediatric like approach;
- Peg-asparaginase-toxicity;
- Risk factors.

During a second phase, the questionnaire was validated by 10 external clinicians and then submitted to a panel of 19 additional Italian clinical experts in the field, through an online platform for a first Delphi round. The results of the questionnaire were then analysed by the board, which re-formulated the items that were not sufficiently well categorized during the first round (*n* = 2). For these two items a second Delphi round was performed using the revised version of items.

### Delphi questionnaire

As reported in detail in the supplementary file 1, the Delphi questionnaire contained 9 statements, each containing three to six items: 1) clinician’s attitudes on Peg-ASP; 2) toxicity profile; 3) hypersensitivity reactions; 4) hepatic toxicity; 5) hepatic and/or metabolic toxicity; 6) haemorrhagic/thrombotic toxicity; 7) pancreatitis; 8) metabolic toxicity management and prevention; 9) activity profile monitoring. The 19 participating experts were invited to express their level of agreement or disagreement on each item using a Likert-type scale from 1 to 5 (1 = strongly disagree, 2 = disagree, 3 = somewhat agree, 4 = agree, 5 = strongly agree). Results were expressed as a percentage of respondents who scored each item as 1 or 2 (disagreement) or as 3, 4 or 5 (agreement). Consensus was achieved when the sum for disagreement or agreement was ≥66%: affirmative consensus was defined in case of agreement ≥66%, negative consensus in case of disagreement ≥66%, while when the sum for disagreement or agreement was below 66% consensus was not reached.

## Results

**Fig. 1:**
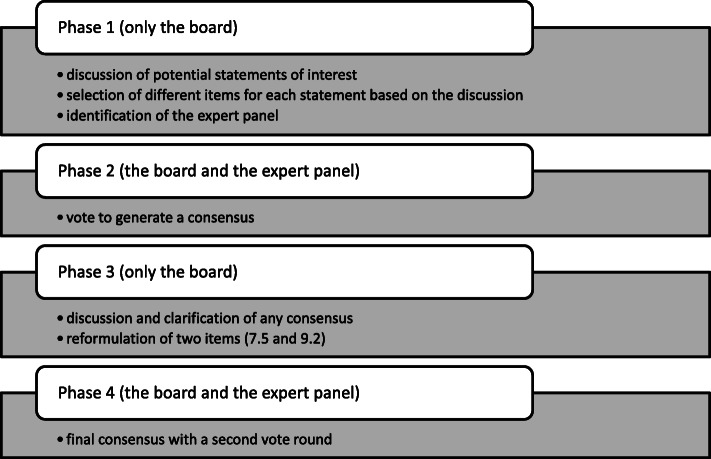
Flow chart of Delphi process

Among all items, 9 did not reach consensus, 29 reached a positive consensus and 3 achieved a negative consensus. The board decided to reformulate items 7.5 and 9.2 to improve clarity and avoid confusion in interpreting the questions by the expert panel. The revised version of item 7.5 reached an affirmative consensus, while item 9.2 did not reach any consensus. The Delphi process is outlined in Fig. 1. The final consensus for each item is summarized in Table 1.

**Table 1:** Statements and results of the Delphi consensus process

No	Statement	Consensus degree (%)
**1. Clinical usefulness of E.coli Peg-ASP for the treatment of adult patients with Ph negative ALL**		
1.1	Peg-ASP is a pivotal medication in the treatment of ALL, indispensable if the treatment aim is curative	100% affirmative
1.2	There are patients for whom there is an absolute contraindication to the use of Peg-ASP (age, comorbidity)	Consensus not reached
1.3	The study of patient-associated risk factors may significantly limit the incidence of adverse events related to the use of Peg-ASP	100% affirmative
1.4	The toxicities associated with the administration of Peg-ASP are manageable in the majority of adult patients	100% affirmative
**2. Peg-asparaginase toxicity profile**		
2.1	It is difficult to predict and characterize by significant variability from patient to patient (individual predisposition	Consensus not reached
2.2	Appropriate knowledge is needed of the mechanisms underlying the development of toxicities in order to establish adequate preventive measures and early intervention at the onset of toxicity	100% affirmative
2.3	It may potentially be influenced by concomitant therapies (chemotherapy, antibiotics, antifungals, steroids)	95% affirmative
2.4	Fatal outcome on rare occasions	100% affirmative
**3. Hypersensitivity reactions**		
3.1	It is advisable to pre-medicate every administration of Peg-ASP to reduce its incidence and/or severity	74% affirmative
3.2	In case of known grade 3–4 allergic reaction, further administrations of Peg-ASP are contraindicated	95% affirmative
3.3	In case of established grade 3–4 allergic reaction to Peg-ASP, substitution with the Erwinia chrysanthemi formulation is indicated	95% affirmative
3.4	In case of a clinically manifested hypersensitivity reaction, ASP activity should be measured to promptly identify any possible inactivation of the medication	74% affirmative
**4. Hepatic toxicity**		
4.1	It is the most frequent toxicity and therefore requires close monitoring (pre-during-post-therapy)	95% affirmative
4.2	In addition to abdominal ultrasound, other instrumental examinations are recommended prior to initiating Peg-ASP therapy	Consensus not reached
4.3	BMI > 30 and pre-existing hepatic steatosis contraindicate the use of Peg-ASP	89% negative
4.4	BMI > 30 and pre-existing hepatic steatosis require a reduction in the dosage of Peg-ASP	89% affirmative
4.5	Regardless of severity and degree of compensation, a diagnosis of chronic liver disease is an absolute contraindication of Peg-ASP treatment	74% negative
4.6	Development of grade 3–4 toxicity does not contraindicate subsequent administrations of Peg-ASP	68% affirmative
**5. Treatment of hepatic and/or metabolic toxicity**		
5.1	Concomitant therapy (chemotherapy, antibiotics, antifungals, steroids, other) plays a decisive role in increasing the risk of hepatotoxicity during Peg-ASP therapy	95% affirmative
5.2	L-carnitine is recommended in the event of hyperbilirubinemia	89% affirmative
5.3	Hyperglycaemia should only be corrected with insulin therapy	84% affirmative
5.4	Hypoalbuminemia should be corrected	95% affirmative
**6. Hemorrhagic/thrombotic toxicity**		
6.1	Laboratory alterations of the hemocoagulative parameters in the absence of clinical signs of thrombosis or bleeding do not necessitate discontinuation of Peg-ASP	100% affirmative
6.2	The use of fresh plasma to correct hypofibrinogenemia is not recommended in the absence of haemorrhagic symptoms	89% affirmative
6.3	Prophylaxis with LMWH is always recommended	consensus not reached
6.4	Any concomitant oral contraceptives or hormone replacement therapy should be discontinued	95% affirmative
6.5	It is advisable to correct hypofibrinogenemia with cryoprecipitate	79% affirmative
6.6	Replenishment of AT is advisable to maintain levels consistently above 60%	100% affirmative
**7. Peg asparaginase-associated pancreatitis**		
7.1	Therapy should be discontinued if asymptomatic pancreatitis develops (CTCAE grade 2, i.e. enzymes > 3 times normal or radiological evidence)	Consensus not reached
7.2	The dosage should be reduced if asymptomatic pancreatitis develops (CTCAE grade 2, i.e. enzymes > 3 times normal or radiological evidence)	Consensus not reached
7.3	CTCAE grade 2 pancreatitis, once resolved, does not contraindicate subsequent administration of Peg-ASP	95% affirmative
7.4	Development of CTCAE grade 2 pancreatitis contraindicates subsequent administrations even with a different ASP formulation (Erwinia chrysanthemi)	79% negative
7.5	Development of CTCAE grade 3–4 pancreatitis contraindicates subsequent administrations even with a different ASP formulation (Erwinia chrysanthemi)	95% affirmative
**8. Metabolic toxicity management and prevention**		
8.1	In the event of hyperglycemia with Peg-ASP and steroid therapy, it may be appropriate to reduce the steroid dose and enhance the insulin therapy rather than delay subsequent administrations of Peg-ASP	100% affirmative
8.2	Patients being treated should be monitored for triglycerides	100% affirmative
8.3	In case of severe (> 500 mg/dl), persistent, isolated hypertriglyceridemia, it is advisable to delay subsequent administrations of ASP	Consensus not reached
8.4	There is insufficient evidence that reducing the Peg-ASP dose reduces development of hepatopancreatic, thrombotic and metabolic toxicity	Consensus not reached
8.5	A preventive reduction in the dosage is always advisable if factors predisposing the development of toxicity are identified (e.g. BMI > 30, hepatosteatosis)	84% affirmative consensus
**9. Monitoring of asparaginase plasmatic activity**		
9.1	Monitoring of asparaginase plasmatic activity is essential in clinical practice in order to optimize the therapeutic activity of asparaginase (e.g. change of formulation in case of drug inactivation)	74% affirmative
9.2	In clinical practice, routine monitoring of ASP plasmatic activity is useful in all patients with ALL	Consensus not reached
9.3	Searching for anti-asparaginase antibodies is of questionable value and should not therefore be routinely performed outside research studies in adult ALL	74% affirmative

### Consensus statements

1. *Clinical usefulness of E.coli* Peg-ASP *for the treatment of adult patients with Philadelphia negative acute lymphoblastic leukemia*
  1.1 Peg-ASP is a pivotal medication in the treatment of ALL, indispensable if the treatment aim is curative. Consensus result: 100% affirmative, median score 5 (range 3–5)
  1.2 There are patients for whom there is an absolute contraindication to the use of Peg-ASP (age, comorbidity). Consensus result: consensus not reached, median score 3 (range 2–5)
  1.3 The study of patient-associated risk factors may significantly limit the incidence of adverse events related to the use of Peg-ASP). Consensus result: 100% affirmative, median score 4 (range 3–5)
  1.4 The toxicities associated with the administration of Peg-ASP are manageable in the majority of adult patients. Consensus result: 100% affirmative, median score 4 (range 3–5)
2. *Peg-asparaginase toxicity profile*
  2.1 It is difficult to predict and characterise the Peg-ASP toxicity profile due to inter-patient significant variability (individual predisposition). Consensus result: consensus not reached, median score 3 (range 1–4)
  2.2 It is needed an appropriate knowledge of the mechanisms underlying the development of toxicities in order to establish adequate preventive measures and early intervention at the onset of toxicity. Consensus result: 100% affirmative, median score 4 (range 3–5)
  2.3 Concomitant therapies (chemotherapy, antibiotics, antifungals, steroids) may potentially influence the toxicity profile. Consensus result: 95% affirmative, median score 4 (range 2–5)
  2.4 Fatal outcome on rare occasions. Consensus result: 100% affirmative, median score 4 (range 3–5)
3. *Hypersensitivity reactions*
  3.1 It is advisable to pre-medicate with hydrocortisone 100 mg intravenous (IV) every administration of Peg-ASP to reduce its incidence and/or severity. Consensus result: 74% affirmative consensus, median score 4 (range 2–5)
  3.2 In case of known grade 3–4 allergic reaction, further administrations of Peg-ASP are contraindicated. Consensus result: 95% affirmative, median score 4 (range 2–5)
  3.3 In case of established grade 3–4 allergic reaction to Peg-ASP, substitution with the Erwinia chrysanthemi formulation is indicated. Consensus result: 95% affirmative, median score 4 (range 2–5)
  3.4 In case of a clinically manifested hypersensitivity reaction, ASP activity should be measured to promptly identify any possible inactivation of the medication. Consensus result: 74% affirmative consensus, median score 3 (range 2–5).
4. *Hepatic toxicity*
  4.1 It is the most frequent toxicity and therefore requires close monitoring (pre-during-post-therapy). Consensus result: 95% affirmative, median score 4 (range 2–5)
  4.2 In addition to abdominal ultrasound, additional instrumental examinations are recommended prior to initiating Peg-ASP therapy. Consensus result: consensus not reached, median score 2 (range 1–5)
  4.3 BMI > 30 and pre-existing hepatic steatosis contraindicate the use of Peg-ASP. Consensus result: 89% negative, median score 2 (range 1–3)
  4.4 BMI > 30 and pre-existing hepatic steatosis require a reduction in the dosage of Peg-ASP. Consensus result: 89% affirmative, median score 4 (range 2–5)
  4.5 Regardless of severity and degree of compensation, a diagnosis of chronic liver disease is an absolute contraindication of Peg-ASP treatment. Consensus result: 74% negative, median score 2 (range 1–5)
  4.6 Development of grade 3–4 toxicity does not contraindicate subsequent administrations of Peg-ASP. Consensus result: 68% affirmative, median score 3 (range 1–4)
5. *Treatment of hepatic and/or metabolic toxicity*
  5.1 Concomitant therapy (chemotherapy, antibiotics, antifungals, steroids, other) plays a decisive role in increasing the risk of hepatotoxicity during Peg-ASP therapy. Consensus result: 95% affirmative consensus, median score 4 (range 2–5)
  5.2 L-carnitine is recommended in the event of hyperbilirubinemia. Consensus result: 89% affirmative consensus, median score 4 (range 2–5)
  5.3 Hyperglycemia should only be corrected with insulin therapy. Consensus result: 84% affirmative consensus, median score 4 (range 2–5)
  5.4 Hypoalbuminemia should be corrected. Consensus result: 95% affirmative consensus, median score 4 (range 2–5)
6. *Hemorrhagic/thrombotic toxicity*
  6.1 Laboratory alterations of the hemocoagulative parameters in the absence of clinical signs of thrombosis or bleeding do not necessitate discontinuation of Peg-ASP. Consensus result: 100% affirmative consensus, median score 5 (range 3–5)
  6.2 The use of fresh plasma to correct hypofibrinogenemia is not recommended in the absence of haemorrhagic symptoms. Consensus result: 89% affirmative consensus, median score 4 (range 2–5)
  6.3 Prophylaxis with low molecular weight heparin (LMWH) is always recommended. Consensus result: consensus not reached, median score 3 (range 1–5)
  6.4 Any concomitant oral contraceptives or hormone replacement therapy should be discontinued. Consensus result: 95% affirmative, median score 4 (range 2–5)
  6.5 It is advisable to correct hypofibrinogenemia with cryoprecipitate. Consensus result: 79% affirmative, median score 4 (range 2–5)
  6.6 Replenishment of antithrombin (AT) is advisable to maintain levels consistently above 60%. Consensus result: 100% affirmative consensus, median score 5 (range 3–5)
7. *Peg asparaginase-associated pancreatitis*
  7.1 Therapy should be discontinued if asymptomatic pancreatitis develops (CTCAE grade 2, i.e. enzymes > 3 times normal or radiological evidence). Consensus result: consensus not reached, median score 2 (range 1–5)
  7.2 The dosage should be reduced if asymptomatic pancreatitis develops (CTCAE grade 2, i.e. enzymes > 3 times normal or radiological evidence). Consensus result: consensus not reached, median score 3 (range 1–5)
  7.3 CTCAE grade 2 pancreatitis, once resolved, does not contraindicate subsequent administration of Peg-ASP. Consensus result: 95% affirmative consensus, median score 4 (range 2–5)
  7.4 Development of CTCAE grade 2 pancreatitis contraindicates subsequent administrations even with a different ASP formulation (Erwinia chrysanthemi). Consensus result: 79% negative consensus, median score 2 (range 1–3)
  7.5 Development of CTCAE grade 3–4 pancreatitis contraindicates subsequent administrations even with a different ASP formulation (Erwinia chrysanthemi). Consensus result: 95% consensus, median score 4 (range 2–5)
8. *Metabolic toxicity management and prevention*
  8.1 In the event of hyperglycemia with Peg-ASP and steroid therapy, it may be appropriate to reduce the steroid dose and enhance the insulin therapy rather than delay subsequent administrations of Peg-ASP. Consensus result: 100% affirmative, median score 4 (range 3–5)
  8.2 Patients being treated should be monitored for triglycerides. Consensus result: 100% affirmative, median score 4 (range 3–5)
  8.3 In case of severe (> 500 mg/dl), persistent, isolated hypertriglyceridemia, it is advisable to delay subsequent administrations of ASP. Consensus result: consensus not reached, median score 3 (range 1–5)
  8.4 There is insufficient evidence that reducing the Peg-ASP dose reduces development of hepatopancreatic, thrombotic and metabolic toxicity. Consensus result: consensus not reached, median score 3 (range 1–4)
  8.5 A preventive reduction in the dosage is always advisable if factors predisposing the development of toxicity are identified (e.g. BMI > 30, hepatosteatosis). Consensus result: 84% affirmative consensus, median score 4 (range 2–5)
9. *Monitoring of asparaginase plasmatic activity*
  9.1 Monitoring of asparaginase plasmatic activity is essential in clinical practice in order to optimize the therapeutic effects of asparaginase (e.g. change of formulation in case of drug inactivation). Consensus result: 74% affirmative consensus, median score 3 (range 2–5)
  9.2 In clinical practice, routine monitoring of ASP plasmatic activity is useful in all patients with ALL. Consensus result: consensus not reached, median score 3 (range 2–4)
  9.3 Searching for anti-asparaginase antibodies is of questionable value and should not therefore be routinely performed outside research studies in adult ALL. Consensus result: 74% affirmative consensus, median score 3 (range 1–5)

## Discussion

The complete consensus on the need of using Peg-ASP in the treatment of ALL reflects the fact that this drug is considered essential to improve treatment results. In this regard, in childhood ALL several trials have clearly demonstrated the benefits from intensified ASP therapy [29–32] and also in adult patients there is an increasing evidence of an improved result in ASP-treated vs no-ASP-treated patients [6, 33–35], as exemplified in the large German Multicenter Study Group for Adult (GMALL) 07/2003 study [7]. Overall, in this study, Peg-ASP intensification resulted feasible in the majority of patients although a higher incidence of severe hepatic toxicity was reported.

In contrast, uncertainty remains over the absolute contraindication to the treatment with Peg-ASP. Participants agreed that special attention is required to identify patients with significant risk factors of toxicity that may outweigh the benefit of treatment.

There was consensus that hepatic toxicity is the most common complication in adult patients receiving Peg-ASP [36]. Clinicians agreed that in the presence of BMI > 30 and pre-existing hepatic steatosis a reduction of Peg-ASP dosage is required. Higher age, obesity (body mass index [BMI] > 30) are indeed established risk factors for the development of severe hepatic toxicity) [36, 37]. Acute onset of hepatic steatosis (with anatomo-pathological features of micro-vesicular steatosis) is a common consequence of ASP treatment [38, 39], so pre-existing hepatic steatosis should be considered a warning for the development of ASP-related hepatic toxicity. As a general rule, a diagnosis of chronic liver disease does not represent an absolute contraindication for Peg-ASP treatment, but in these patients the board suggested to consider the use of Peg-ASP on an individual basis and after careful evaluation of its clinical severity. In case of hepatic toxicity, also up to grade 3–4, occurring during treatment with Peg-ASP, subsequent administrations are still indicated after delaying the treatment until AST/ALT levels and bilirubin are within 5xULN and 3xULN ranges, respectively [40]. An example on how Peg-ASP treatment could be modulated based on the severity of the toxicity pattern and on the number of previous exposures is the algorithm currently adopted in the ongoing GIMEMA LAL 2317 trial [24]. Although this approach is not yet validated by clinical results, it represents a first attempt to introduce a risk- and age-oriented Peg-ASP dosing in an induction/consolidation multi-agent protocol carrying substantial risks of hematological and extra-hematological toxicity. A near complete consensus was obtained on the fact that concomitant therapies play a major role in determining hepatotoxicity during treatment with Peg-ASP. Therefore, preventive treatment strategies to decrease hepatotoxicity should include avoiding or adjusting the concurrent use of hepatotoxic drugs, including azol antifungal and antibiotics [40]. In addition, in the future protocols a careful spacing in terms of days before the use of other hepatotoxic antileukemic agents (anthracyclines, corticosteroids, cyclophosphamide and antimetabolites) should be taken into account.

There was consensus that, although Peg-ASP results in less immunogenicity than the native compound [9], pre-medication of each Peg-ASP dose with hydrocortisone 100 mg IV is an essential step in order to reduce the likelihood and/or severity of acute hypersensitivity reaction. This consensus is in keeping with suggestions provided by an international expert panel [40]. Almost all clinicians who responded to the survey agreed that in case of serious allergic reactions (grade 3–4 according to National Cancer Institute (NCI) CTCAE) permanent discontinuation of Peg-ASP is indicated. Peg-ASP displays cross-reactivity with native *E. coli* ASP and allergic reactions are often also associated with an inactivation of the drug due to the development of ASP antibodies [11–13, 30]. In this regard, clinicians indicated that in patients with serious allergic reaction a substitution with the *Erwinia chrysantemi* formulation is a preferable alternative to reduce the risk of subsequent allergic reactions and to ensure continued depletion of asparagine. Indeed, crisantaspase does not display cross-reactivity to either of the *E. coli*-derived products [41]. A weaker consensus was reached by the panel on the need of serum ASP activity level assessment in order to recognize a suboptimal enzyme activity, generally due to the formation of neutralizing antibodies, in patients with allergic reaction. This uncertainty reflects the logistic limitations in measuring ASP activity levels [42, 43] which made this strategy impractical for a broad clinical use in past Italian trials. Currently monitoring of ASP activity is available on request within the GIMEMA study ALL2518 [44].

The use of ASP is associated with reduced insulin production and possibly a decrease in the expression of insulin receptors [45, 46]. In addition, corticosteroids cause insulin resistance. Therefore, hyperglycemia is quite common during phases of therapy with concomitant use of Peg-ASP and corticosteroids. Clinicians of the panel believe that safe management of hyperglycemia requires only the use of insulin. The use of oral hypoglycemic drugs should be avoided because they may increase the risk of hepatotoxicity.

In case of hyperbilirubinemia the use of L-carnitine is recommended as suggested in some reports [47–49]. A suggested L-carnitine schedule is 50 mg/kg daily intravenously in divided doses for 5–8 days until toxicity resolves. Addition of vitamin B complexes may also be helpful. Finally, hypo-albumin should be corrected by infusion of serum albumin, with the aim to maintain albumin levels ≥2.5 g/dl, in order to prevent peripheral edema and/or the more severe condition of anasarca. Maintaining a good albumin level is also essential to provide an adequate carrier mechanism to several antibacterial agents employed in case of neutropenic infectious complications. Of note, in the absence of studies that directly examine the benefit of correcting hypoalbuminemia, this suggestion derives from the formal consent of the expert panel.

The panel agrees not to withhold treatment for abnormal coagulation tests without clinical correlates. Although the benefit of correcting hypofibrinogenemia in terms of preventing hemorrhagic complications is unclear both in pediatric and adult setting [50, 51], the expert panel considered cost-effective to correct hypofibrinogenemia with the use of cryoprecipitate. A consensus was reached against the use of fresh frozen plasma (FFP) since FFP may replete serum asparagine counteracting the anti-leukemic effect of ASP. In addition, the use of fibrinogen concentrates should be avoided in the absence of haemorrhagic symptoms since they may increase the risk of thrombosis [51].

A complete consensus on the need of antithrombin infusion if antithrombin level < 60% was obtained. This reflects the hypothetical linking between ASP-induced antithrombin deficiency and the risk of developing thrombotic events [52]. In this regard, a meta-analysis of 17 studies focusing on thrombotic complications in children with ALL found that the overall incidence of thrombosis is 5.2% and that slightly more than the 50% of the events occurred in the central nervous system (CNS), and 28.6% of the total events were classified as cerebral venous thrombosis [53]. Considering the higher risk of thrombotic events associated with the use of female hormones [54, 55] and the above-mentioned risk of thrombosis related to ASP-induced antithrombin deficiency, the panel suggested to avoid any concomitant hormonal therapies during treatment with Peg-ASP. The impact of heparin prophylaxis remains controversial [51, 56]. The results of a randomized study in the pediatric setting showed that prophylactic use of enoxaparin significantly reduced thromboembolism during induction therapy [56]. In contrast a very recent observational study in the adults setting by the French group GRAALL showed that the use of heparin prophylaxis was associated with a surprisingly increased risk of thrombosis [51]. In this context of uncertainty, a consensus was not reached about the use of primary antithrombotic prophylaxis with LMWH, that mainly reflects the perception of a concomitant high bleeding risk for these patients, both due to a decrease in the production of proteins involved in coagulation associated with ASP use and to the relevant thrombocytopenia, usually observed during induction and consolidation cycles.

Some discrepancies exist concerning the use of Peg-ASP in patients with chemical pancreatitis (e.g. elevation of amylase and/or lipase). The recommendations of an international expert panel reported to continue Peg-ASP treatment [40], considering that this form of toxicity is usually not life-threatening and resolves without long-term sequelae [57, 58]. In patients with grade 2 pancreatitis, daily monitoring of enzymes is required and the drug should be withheld until amylase and lipase levels are below 3 times the upper limit of normal (ULN), in the absence of clinical symptoms [40]. There was a near complete consensus that in case of grade ≥ 3 pancreatitis subsequent administration of ASP is contraindicated. Since the formulation of L-ASP does not influence the incidence of pancreatitis [59], the use of a different formulation (Erwinia chrysanthemi) is also contraindicated.

There is uncertainty if a transient discontinuation of Peg-ASP is appropriate in patients developing isolated severe hypertriglyceridemia (≥ 500 mg/dl). A recent study showed that a transient elevation in triglyceride levels of grade 3/4 was seen in about 47% of patients receiving ASP and corticosteroids and it is usually asymptomatic [60]. These patients should be closely monitored for signs of pancreatitis [60, 61], while there is not currently a specific treatment for hypertriglyceridemia. Hypertriglyceridemia is generally temporary and levels return to normal completely after Peg-ASP treatment is concluded [60]. In the rare cases displaying very high levels of triglycerides (i.e. ≥ 2000 mg/dL) a temporary discontinuation of Peg-Asp may be prudential. Hyperidratation and omega-3 fatty acids supplementation can accelerate the restoration of normal triglycerides levels. Based on the above-mentioned data, clinicians agreed that checking triglycerides levels in patients receiving Peg-ASP is useful, so that patients with severe hypertriglyceridemia at higher risk of developing pancreatitis or for whom a temporary discontinuation of treatment might be indicated are more easily identified.

There is an increasing agreement that monitoring of plasma ASP activity may be relevant in clinical practice in order to ascertain adequate ASP efficacy, since plasma activity levels > = 0.1 IU/ml correspond to a profound asparagine depletion and to an adequate therapeutic effect [11, 30, 62]. Another relevant advantage of measuring plasma ASP activity levels is the possibility to optimize the therapeutic effects of ASP (e.g. change formulation in case of inactivation of the drug) [63]. A consensus was not reached about the use of routine monitoring of ASP activity in clinical practice. However, its use should be considered for screening selected patients with overt or even doubtful signs of hypersensitivity [11, 43]. Indeed, this information may be of great value in clinical decision-making of switching to a different ASP preparation to ensure continuous plasma depletion of asparagine [12, 13, 63, 64]. Screening patients for neutralizing anti-asparaginase antibodies has been suggested in some reports, but the specificity of anti-asparaginase antibodies to predict inactivation has been found to be low compared with measuring ASP activity itself [43]. The measurement of asparaginase activity (> 0.1 IU/ml) remains the most direct and convincing assessment of asparaginase effectiveness. In this regard, most recent data should be taken into account. In a pediatric study using a more sensitive technique, an effective serum asparagine depletion was obtained with an enzymatic activity as low as 0.02 IU/ml [65]; also, in adult patients, 80–90% of the cases receiving Peg-ASP 500–1000 IU/m^2^ reached drug levels > 0.1 IU/ml, that persisted for 14 days in 77% with 1000 IU/m^2^ and for 7 days in 59% with 500 IU/m^2^ [66]. Finally, a recent study reported that adults with comorbidities receiving a reduced dose of PEG-ASP < 1000 IU/m^2^ experienced fewer toxicities while still attaining therapeutic activity levels > 0.1 IU/mL [67]. The final consensus of the panel against the utility of routinely searching anti-asparaginase antibodies reflects the data mentioned above.

## Conclusions

The development of pediatric-inspired treatment protocols for adult ALL has led to an increasing use of PegAsp in adults. The relatively high incidence of drug-related adverse events has prompted a need for suggestions in the management and prevention of toxicities. The most serious adverse effects associated with ASP in adult ALL patients are hepatotoxicity, pancreatitis, hyperglycemia and hypertriglyceridemia, thrombosis, alterations of the hemocoagulative parameters and hypersensitivity. Here we reported the results of a Delphi consensus by a group of Italian experts in treating adult ALL that highlights the importance of a careful monitoring of drug toxicity and a correct evaluation of risk factors for each patient in order to reduce the risk of adverse events. Similar to the empirical guidelines provided in the most recent GIMEMA trial [24], the experts suggested that PegAsp schedule and dosing should take into account patient’s age, body mass index and liver steatosis and whether or not grade 3–4 drug toxicity during first or prior Peg-ASP exposure has been observed. Coagulopathy (antithrombin and/or fibrinogen deficiency) can be prevented by the periodic infusion of antithrombin and cryoprecipitate as needed. The use of L-carnitine should be considered in patients with severe liver injury with direct bilirubin > 3 mg/d/L. Finally, although Peg-ASP results in less immunogenicity than the native compound, pre-medication of each Peg-ASP dose with hydrocortisone 100 mg IV is advisable in order to reduce the likelihood and/or severity of acute hypersensitivity reaction. Moreover, therapeutic dose monitoring of Asp activity levels should be implemented to identify patients with suboptimal activity levels to adjust treatment accordingly. A summary of the main advices in the management of Peg-ASP toxicity are reported in Table 2.

**Table 2:** Summary of the main advices from Delphi questionnaire in the management of Peg-ASP toxicity

No	Prevention and Management
**1. Hypersensitivity reactions**	
	It is advisable to pre-medicate every administration of Peg-ASP to reduce incidence and/or severity of hypersensitivity reactions
	In case of known grade 3–4 allergic reaction, further administrations of Peg-ASP are contraindicated and substitution with the Erwinia chrysanthemi formulation is indicated
	In case of a clinically manifested hypersensitivity reaction, ASP activity should be measured to promptly identify any possible inactivation of the medication
**2. Hepatic toxicity**	
	BMI > 30 and pre-existing hepatic steatosis require a reduction in the dosage of Peg-ASP
	Development of grade 3–4 toxicity does not contraindicate subsequent administrations of Peg-ASP when grade toxicity ≤2
	L-carnitine is suggested in the event of hyperbilirubinemia
	Concomitant therapy (chemotherapy, antibiotics, antifungals, steroids, other) plays a decisive role in increasing the risk of hepatotoxicity during Peg-ASP therapy
**3. Metabolic toxicity**	
	Hyperglycaemia should be corrected with insulin therapy and it is not an indication to discontinue or delay subsequent PegAsp administrations
	Hypoalbuminemia should be corrected
**4. Hemorrhagic/thrombotic toxicity**	
	Laboratory alterations of the hemocoagulative parameters in the absence of clinical signs of thrombosis or bleeding do not necessitate discontinuation or delay of Peg-ASP
	It is advisable to correct hypofibrinogenemia with cryoprecipitate
	Replenishment of AT is advisable to maintain levels consistently above 60%
**5. Peg asparaginase-associated pancreatitis**	
	CTCAE grade 2 asymptomatic pancreatitis, once resolved, does not contraindicate subsequent administration of Peg-ASP
	Development of symptomatic pancreatitis or asymptomatic with CTCAE grade 3–4 amylase/lipase elevation contraindicates subsequent administrations even with a different ASP formulation

## Supplementary information

**Additional file 1:.** List of involved experts.

**Additional file 2:.** Delphi questionnaire.

## Data Availability

The dataset used and analysed during the current study is available from the corresponding author on reasonable request.

## Abbreviations

ALT: Alanine aminotransferase
apt: Activated partial thromboplastin time
AST: Aspartate aminotransferase
AYA: Adolescents and young adults
ASP: Asparaginase
ALL: Acute lymphoblastic leukemia
BMI: Body mass index
CNS: Central nervous system
CTCAE: Common Terminology Criteria for Adverse Events
FDA: Food and drug administration
FFP: Fresh frozen plasma
GMALL: German Multicenter Study Group for Adult
GIMEMA: Gruppo Italiano Malattie Ematologiche dell’Adulto
INR: International normalized ratio
IV: Intravenous
IU: International unit
LLN: Lower limit of normal
LMWH: Low molecular weight heparin
Ml: Milliliter
NCI: National Cancer Institute
Peg-ASP: Pegylated asparaginase
Ph-: Philadelphia-negative
ULN: Upper limit of normal
