# Supplementary material for: National Italian Delphi panel consensus: which measures are indicated to minimize pegylated-asparaginase associated toxicity during treatment of adult acute lymphoblastic leukemia?
Source: BMC Cancer. 2020 Oct 2;20:956. doi: 10.1186/s12885-020-07461-5 (PMC7532578; doi:10.1186/s12885-020-07461-5)
Supplement: Supplementary file 1 — Additional file 1:. List of involved experts. [file 12885_2020_7461_MOESM1_ESM.docx]

**Supplementary information**

List of involved experts:

1.           Audisio Ernesta – Torino

2.           Ballerini Filippo - Genova

3.           Bigazzi Catia – Ascoli Piceno

4.           Bonifacio Massimiliano - Verona

5.           Carluccio Paola – Bari

6.           Carobolante Francesca – Mestre

7.           De Fabritiis Paolo – Roma

8.           Finizio Olimpia – Napoli

9.           Guolo Fabio – Genova

10.         Krampera Mauro – Verona

11.         Lanza Francesco – Ravenna

12.         Mauro Elisa – Catania

13.         Minotti Clara – Roma

14.         Rambaldi Alessandro - Bergamo

15.         Rossi Marianna - Milano

16.         Scattolin Anna Maria – Mestre

17.         Tosi Patrizia - Rimini

18.         Zappasodi Patrizia – Pavia

19.         Zappone Elisabetta – Siena
