# Supplementary material for: National Italian Delphi panel consensus: which measures are indicated to minimize pegylated-asparaginase associated toxicity during treatment of adult acute lymphoblastic leukemia?
Source: BMC Cancer. 2020 Oct 2;20:956. doi: 10.1186/s12885-020-07461-5 (PMC7532578; doi:10.1186/s12885-020-07461-5)
Supplement: Supplementary file 2 — Additional file 2:. Delphi questionnaire. [file 12885_2020_7461_MOESM2_ESM.docx]

**Delphi questionnaire**

1 Regarding the use of E.coli Peg-asparaginase for the treatment of adult patients with Philadelphia negative acute lymphoblastic leukaemia, I consider that:

1.1 Peg-ASP is a pivotal medication in the treatment of ALL, indispensable if the treatment aim is curative

1.2 There are patients for whom there is an absolute contraindication to the use of Peg-ASP (age, comorbidity)

1.3 The study of patient-associated risk factors may significantly limit the incidence of adverse events related to the use of Peg-ASP)

1.4 The toxicities associated with the administration of Peg-ASP are manageable in the majority of adult patients

2 With regard to Peg-asparaginase toxicity profile, I consider that:

2.1 It is difficult to predict and characterise the Peg-ASP toxicity profile due to inter-patient significant variability (individual predisposition)

2.2 It is needed an appropriate knowledge of the mechanisms underlying the development of toxicities in order to establish adequate preventive measures and early intervention at the onset of toxicity

2.3 Concomitant therapies (chemotherapy, antibiotics, antifungals, steroids) may potentially influence the toxicity profile

2.4 Fatal outcome on rare occasions

3 Regarding hypersensitivity reactions, I consider that:

3.1 It is advisable to pre-medicate every administration of Peg-ASP to reduce its incidence and/or severity

3.2 In case of known grade 3-4 allergic reaction, further administrations of Peg-ASP are contraindicated

3.3 In case of established grade 3-4 allergic reaction to Peg-ASP, substitution with the Erwinia chrysanthemi formulation is indicated

3.4 In case of a clinically manifested hypersensitivity reaction, ASP activity should be measured to promptly identify any possible inactivation of the medication

4 Regarding hepatic toxicity of Peg-asparaginase, I consider that:

4.1 It is the most frequent toxicity and therefore requires close monitoring (pre-during-post-therapy)

4.2 In addition to abdominal ultrasound, additional instrumental examinations are recommended prior to initiating Peg-ASP therapy

4.3 BMI >30 and pre-existing hepatic steatosis contraindicate the use of Peg-ASP

4.4 BMI >30 and pre-existing hepatic steatosis require a reduction in the dosage of Peg-ASP

4.5 Regardless of severity and degree of compensation, a diagnosis of chronic liver disease is an absolute contraindication of Peg-ASP treatment

4.6 Development of grade 3-4 toxicity does not contraindicate subsequent administrations of Peg-ASP

5 Regarding treatment of hepatic and/or metabolic toxicity of Peg-asparaginase, I consider that:

5.1 Concomitant therapy (chemotherapy, antibiotics, antifungals, steroids, other) plays a decisive role in increasing the risk of hepatotoxicity during Peg-ASP therapy

5.2 L-carnitine is recommended in the event of hyperbilirubinemia

5.3 Hyperglycemia should only be corrected with insulin therapy

5.4 Hypoalbuminemia should be corrected

6 Regarding hemorrhagic/thrombotic Peg-asparaginase toxicity, I consider that:

6.1 Laboratory alterations of the hemocoagulative parameters in the absence of clinical signs of thrombosis or bleeding do not necessitate discontinuation of Peg-ASP

6.2 The use of fresh plasma to correct hypofibrinogenemia is not recommended in the absence of haemorrhagic symptoms

6.3 Prophylaxis with low molecular weight heparin (LMWH) is always recommended

6.4 Any concomitant oral contraceptives or hormone replacement therapy should be discontinued. 6.5 It is advisable to correct hypofibrinogenemia with cryoprecipitate

6.6 Replenishment of antithrombin is advisable to maintain levels consistently above 60%

7 Regarding Peg-asparaginase-associated pancreatitis, I consider that:

7.1 Therapy should be discontinued if asymptomatic pancreatitis develops (CTCAE grade 2, i.e. enzymes > 3 times normal or radiological evidence)

7.2 The dosage should be reduced if asymptomatic pancreatitis develops (CTCAE grade 2, i.e. enzymes > 3 times normal or radiological evidence)

7.3 CTCAE grade 2 pancreatitis, once resolved, does not contraindicate subsequent administration of Peg-ASP

7.4 Development of CTCAE grade 2 pancreatitis contraindicates subsequent administrations even with a different ASP formulation (Erwinia chrysanthemi)

7.5 Development of CTCAE grade 3-4 pancreatitis contraindicates subsequent administrations even with a different ASP formulation (Erwinia chrysanthemi)

8 Regarding the risk of metabolic toxicity management and prevention, I consider that:

8.1 In the event of hyperglycemia with Peg-ASP and steroid therapy, it may be appropriate to reduce the steroid dose and enhance the insulin therapy rather than delay subsequent administrations of Peg-ASP

8.2 Patients being treated should be monitored for triglycerides

8.3 In case of severe (>500 mg/dl), persistent, isolated hypertriglyceridemia, it is advisable to delay subsequent administrations of ASP

8.4 There is insufficient evidence that reducing the Peg-ASP dose reduces development of hepatopancreatic, thrombotic and metabolic toxicity

8.5 A preventive reduction in the dosage is always advisable if factors predisposing the development of toxicity are identified (e.g. BMI >30, hepatosteatosis)

9 Regarding the monitoring of asparaginase plasmatic activity, I consider that:

9.1 Monitoring of asparaginase plasmatic activity is essential in clinical practice in order to optimize the therapeutic effects of asparaginase (e.g. change of formulation in case of drug inactivation)

9.2 In clinical practice, routine monitoring of ASP plasmatic activity is useful in all patients with ALL

9.3 Searching for anti-asparaginase antibodies is of questionable value and should not therefore be routinely performed outside research studies in adult ALL
